# Mercurial Poisoning

**Published:** 1888-04

**Authors:** 


### MERCURIAL POISONING.

TRANSLATED FROM THE "ZEITSCHRIFT FÜR HOMŒOPATHIE," BY DR. A. MCNEIL.

During the session of the Berliner Medicinische Gesellschaft (Berlin Medical Society), of November, 1887, Professor Virchow gave a lecture, illustrated by pathologico-anatomical preparations, on poisoning by corrosive sublimate (Mercurius corr.). He showed that by washing wounds with the now fashionable solution of the sublimate in order to kill the supposed dangerous bacteria, inflammatory conditions of the intestinal mucous membrane were produced. This was more particularly the case in the large intestines, and when thus inflamed it was hardly to be distinguished from dysentery. He was convinced from autopsies recently made that many patients had died, not from dysentery, as supposed, but from poisoning by corrosive sublimate used in the treatment of wounds. These observations were indorsed by Professors Liebreich and Senator.

Every homœopathic physician, since the publication of Hahnemann's *Materia Medica Pura* is acquainted with the fact that the symptoms of dysentery [some varieties—McNeil] are similar to those of the proving of Mercurius corr., and that this sublimate of quicksilver, when homœopathically potentized, is one of the best known and most useful remedies in treating inflammation of the bowels.

These boasting academicians, bloated with "science" and bragging of their "rational" treatment of the sick, might learn much from the "unscientific" homœopaths; might learn at least not to make men sick with their crude experiments at disinfection. The confessions of these distinguished professors prove they do not know how to treat these and other diseased conditions. [How about the "scientific" treatment the poor Crown Prince is now receiving from the most celebrated physicians the "regulars" can boast of? Shall we desert Homœopathy to try such botching?—EDITOR H. P.]

